# Neurodevelopmental Pathways: Between Pathologisation and Neurodiversity

**DOI:** 10.3390/jcm11102753

**Published:** 2022-05-12

**Authors:** Michele Roccella, Luigi Vetri

**Affiliations:** 1 Department of Psychology, Educational Science and Human Movement, University of Palermo, 90128 Palermo, Italy; michele.roccella@unipa.it; 2 Oasi Research Institute-IRCCS, Via Conte Ruggero 73, 94018 Troina, Italy

Accurate identification of children’s pathogenic neuropsychological developmental trajectories or, on the contrary, of children’s typical developmental trajectories is one of the main objectives of developmental psychopathology. This branch of medical science integrates various disciplines and areas of study, including embryology, neuroscience, ethology, clinical psychology, and neuropsychiatry.

The current Special Issue, “10th Anniversary of JCM—Research Updates in Developmental Psychopathology and Paediatric Neurology”, in the Journal of Clinical Medicine, is dedicated to collecting high-quality scientific contributions in neuro–psychopathology—in other words, pathologies of neurological, neuropsychiatric, psychiatric, neuropsychological, and psychological origin, which reduce an individual’s adaptability.

Psychopathological disorders usually begin early during an individual’s neuropsychological development and generally involve some degree of impairment in a child’s personal, social, and academic functioning. Thus, it is essential to pay close attention to the early identification of developmental atypia to ensure timely preventative action. Evidence presented in the scientific literature has shown that children have a strong ability to self-regulate and organise their own perceptual–experiential experience that begins in the first few days of life. The infant builds his early experiences by interacting with the environment, with his caregiver, and his primary care skills, shaping his own reality and influencing activities such as wakefulness, sleep, and nutrition [1].

Analogously, behavioural control mechanisms are closely related to a series of early skills developed in the first few years of life. Behavioural control is closely linked to emotion, attention, and executive function regulation. There is a progressive increase in the child’s abilities to regulate his physiological states. Regulatory processes are in fact based on the ability, present from birth, to regulate one’s emotional states and organise the experience and appropriate behavioural responses, integrating tactile, visual, acoustic, and proprioceptive sensations coming from the internal and external world. However, they are influenced by both mother–child interactions and the child’s temperamental characteristics [2].

The analytical process of the psychopathology of developmental age has to proceed to the early identification of both genetic and environmental psychopathological risk and resilience factors and the analysis of frequent medical and neurological comorbidities that often complicate the clinical picture. In fact, in paediatric neurological diseases such as epilepsy or chronic headaches, psychopathological consequences are common and are sometimes more disabling than the disease itself.

Meticulous clinical and therapeutic characterisation is crucial for effective treatment of the most common childhood psychopathological conditions such as anxiety disorders, mood disorders, attention deficit hyperactivity disorder, autism spectrum disorder, sleep disorders, specific learning disorders, behavioural disorders, intellectual disabilities, eating disorders, tic disorders, and communication disorders.

These disorders are usually “comorbid” with one another, and it is very hard to differentiate between them, especially during the first early diagnostic evaluation, because they share a common genetic predisposition, mutual environmental risk factors, and overlapping clinical symptoms [3].

Those common traits between different neuro–psychopathological conditions complicate attempts at accurate and timely diagnosis. Therefore, Christopher Gillberg proposed overcoming the traditional categorical diagnostic system for early diagnoses of neurodevelopmental disorders. He proposed a completely broader umbrella called ESSENCE: acronym for Early Symptomatic Syndromes Eliciting Neurodevelopmental Clinical Examinations [4].

Indeed, this approach has the merit of more accurately reflecting the clinical reality of neurodevelopmental disorders, especially in the early stages. Indeed, over the years, a dimensional rather than a categorical definition has been chosen for some neurodevelopmental disorders such as autism, ADHD, and schizophrenia.

The main limit of this approach is that it tends to oversize the actual dimension of the problem. Precisely defining the limits of the broader spectrum of neurodevelopmental disorders is very difficult. Effective neuropsychological tools are still lacking to define these limits, and we risk excessive pathologisation of neurodiversity. The hope is that progress in identifying causative, additive, or synergistic genetic variants and the progress of neuroimaging could provide us with additional tools for identifying subjects at risk for neurodevelopmental diseases [5].

This Special Issue will collect the experiences of clinical psychologists, psychiatrists, neurologists, and paediatricians from different areas and countries to acquire new perspectives in the diagnosis and treatment of childhood neurodevelopmental disorders.

Overall, we trust that the articles included in this Special Issue will help to clarify some issues raised in this Editorial, providing further perspectives and greater awareness about the complexity of neurodevelopmental disorders.

## Data Availability

Not applicable.

## References

[1] Benedetto L., Cucinotta F., Maggio R., Germanò E., De Raco R., Alquino A., Impallomeni C., Siracusano R., Vetri L., Roccella M. (2021). One-year follow-up diagnostic stability of autism spectrum disorder diagnosis in a clinical sample of children and toddlers. Brain Sci..

[2] Pastorino G.M.G., Operto F.F., Padovano C., Vivenzio V., Scuoppo C., Pastorino N., Roccella M., Vetri L., Carotenuto M., Coppola G. (2021). Social cognition in neurodevelopmental disorders and epilepsy. Front. Neurol..

[3] Vetri L. (2020). Autism and migraine: An unexplored association?. Brain Sci..

[4] Gillberg C. (2010). The ESSENCE in child psychiatry: Early symptomatic syndromes eliciting neurodevelopmental clinical examinations. Res. Dev. Disabil..

[5] Operto F.F., Pastorino G.M.G., Stellato M., Morcaldi L., Vetri L., Carotenuto M., Viggiano A., Coppola G. (2020). Facial emotion recognition in children and adolescents with specific learning disorder. Brain Sci..

